# Infant vaccination coverage trajectories and immunisation system archetypes in the WHO African region, 2019–2024: a longitudinal analysis of 47 countries

**DOI:** 10.1136/bmjph-2025-004748

**Published:** 2026-10-12

**Authors:** Abdu A Adamu, Aschalew Teka Bekele, Balcha Girma Masresha, Lynda Rey, Sidy Ndiaye, Michael R Adjei, Ado Mpia Bwaka, Rabiu I Jalo, Charles Shey Wiysonge

**Affiliations:** 1 Vaccine-Preventable Diseases Programme, World Health Organization Regional Office for Africa, Brazzaville, Brazzaville, Congo; 2 Ecole Nationale d’Administration Publique - Campus de Montreal, Montreal, Quebec, Canada; 3 Country Office, World Health Organization, Accra, Accra, Ghana; 4 World Health Organization Inter-Country Support West Africa, Ouagadougou, Burkina Faso, Ouagadougou, Burkina Faso; 5 Department of Community Medicine, Bayero University/Aminu Kano Teaching Hospital, Kano, Nigeria; 6 Cochrane South Africa, South African Medical Research Council, Cape Town, Western Cape, South Africa; 7 Stellenbosch University, Cape Town, South Africa

**Keywords:** Public Health, Vaccination, Epidemiology

## Abstract

**Introduction:**

COVID-19 disrupted childhood vaccination, but country-specific heterogeneity in diphtheria–tetanus–pertussis-containing vaccines (DTP3) coverage across the WHO African region remains poorly understood. This study addressed this gap by quantifying temporal trajectories and identifying latent structural patterns in DTP3 coverage across all 47 countries in the WHO African region from 2019 to 2024, spanning the onset and recovery phases of the COVID-19 pandemic.

**Methods:**

Country-level DTP3 coverage data from 2019 to 2024 were extracted from the WHO/United Nations Children’s Fund Estimates of National Immunisation Coverage, published in July 2025. Country-specific ordinary least squares regression models estimated annual rates of change (slopes) in DTP3 coverage, while linear mixed-effects modelling decomposed within-country and between-country variance components. Principal component (PC) analysis (PCA) was then applied to the 6-year coverage matrix to derive two dominant, uncorrelated components capturing coverage level (PC1) and temporal trajectory (PC2). K-means clustering of PC scores grouped countries into empirically distinct immunisation system archetypes.

**Results:**

Between 2019 and 2024, the estimated annual rate of change in DTP3 coverage was positive in 21 countries and negative in 24; Eritrea and Burkina Faso showed no evidence of a linear trend. The fixed-effect slope was 0.07 percentage points per year (95% CI −0.34 to 0.47; p=0.75), indicating no statistically significant net regional change, while between-country heterogeneity was substantial (random-intercept variance, 188.18; random-slope variance, 1.18). PCA showed that PC1 dominated, explaining 93% of variance (capturing sustained coverage level), with PC2 contributing an additional 3% (capturing temporal trajectory). K-means clustering of PC scores yielded four archetypes: cluster 1 (high coverage, consolidating; n=15), cluster 2 (chronically low coverage, divergent trajectories; n=9), cluster 3 (mid-low coverage, predominantly improving; n=11) and cluster 4 (mid-high coverage, erosion risk; n=12).

**Conclusion:**

This study generated an empirically derived, model-driven typology of immunisation system performance across the WHO African region, characterised by four distinct DTP3 coverage archetypes. The typology provides decision-makers with a granular basis for differentiating programmatic strategies, prioritising resource deployment and designing cluster-specific implementation research rather than applying uniform regional solutions.

**WHAT IS ALREADY KNOWN ON THIS TOPIC**

- The COVID-19 pandemic disrupted routine immunisation services globally but with differential impacts on diphtheria–tetanus–pertussis-containing vaccine (DTP3) coverage across countries in the WHO African region. Regional DTP3 coverage declined from 76% in 2019 to 72% in 2022 and then partially recovered to 75% in 2023.

**WHAT THIS STUDY ADDS**

- This study provides the first country-level quantification of DTP3 coverage trajectories across all 47 WHO African region countries over the 2019–2024 period, encompassing COVID-19 disruption and recovery. Using a novel multilayered analytical framework combining linear growth modelling, mixed-effects modelling and principal component analysis–informed clustering, a data-driven typology of four immunisation system archetypes was derived. This typology moves beyond aggregate regional indicators to reveal the hidden structural and dynamic heterogeneity shaping immunisation programme performance across Africa.

**HOW THIS STUDY MIGHT AFFECT RESEARCH, PRACTICE AND POLICY**

- The archetype-based typology enables segmented immunisation policy, allowing national governments, WHO, UNICEF, Africa Centres for Disease Control and Prevention and Gavi, the Vaccine Alliance, to align funding frameworks and programmatic strategies with the specific performance profile of each country cluster. The findings are directly relevant to Gavi Learning, Evidence and Advocacy for Policy (2026–2030) and Immunisation Agenda 2030 implementation planning. The study also establishes an analytical foundation and country-level characteristics to guide future implementation research aimed at explaining differential immunisation progress and testing context-specific innovations.

## Introduction

Vaccination is one of the most effective public health measures for improving child survival worldwide, especially in the WHO African region.^1^ This is because the region has historically borne a disproportionate burden of infectious diseases.^2 3^ A modelling study published in 2024 estimated that vaccination has contributed to a 52% reduction in paediatric mortality in the African region.^1^ Countries within the African region, as well as globally, implement the Expanded Programme on Immunisation (now known as the Essential Programme on Immunisation) to ensure access to nationally recommended vaccines.^1 4 5^ In 2021, the World Health Assembly endorsed an ambitious strategic framework for 2021–2030 called Immunisation Agenda 2030 (IA2030),^6^ which envisions “a world where everyone, everywhere, at every age fully benefits from vaccines for good health and well-being”.

Coverage with three doses of diphtheria–tetanus–pertussis-containing vaccines (DTP3) is a recognised indicator in the IA2030 framework for assessing the capacity of national immunisation programmes to reliably vaccinate children with the recommended series during their first year of life.^7^ Although the African region has persistently remained below the global benchmark of 90% for DTP3, progressive improvement was noted in the pre-COVID-19 period, increasing from 51% in 2000 to 76% in 2019.^8^ With the emergence of COVID-19, which was declared a Public Health Emergency of International Concern (PHEIC) in 2020, widespread health system disruptions occurred across countries.^9^ Since national immunisation programmes rely on health system structures to deliver routine vaccination services, DTP3 coverage notably declined.^8 10^ According to the WHO/UNICEF Estimate of National Immunisation Coverage (WUENIC), DTP3 coverage fell to 74% by 2020 and further decreased to 72% in 2022.^8^ Evidence of recovery only began to emerge in 2023, with DTP3 rising to 75%.^8^ Several countries in the region implemented interventions, such as the Big Catch-Up campaigns, to address vaccination gaps caused by COVID-19.^11 12^ The COVID-19 PHEIC ended in 2023.

Although DTP3 coverage in 2024 has returned to the pre-pandemic level of 76%, it still falls short of the IA2030 target of at least 90%.^8^ Overall, approximately 9.5 million children did not receive DTP3 in 2024 and remain at increased risk of vaccine-preventable diseases.^8^ However, the magnitude of each country’s contribution to this gap varies across the region, underscoring the need to strengthen country-centred action as a programmatic imperative to accelerate DTP3 coverage in Africa. In 2024, only 17 of the 47 countries in the region achieved DTP3 coverage of at least 90%.^8^ For the same year, the countries with the highest numbers of unvaccinated and under-vaccinated children include Nigeria, the Democratic Republic of the Congo, Ethiopia, Angola, Tanzania, Madagascar, Mozambique, South Africa, Chad and Côte d’Ivoire, listed in descending order.^8^ The public health consequences of a protracted gap in vaccination coverage are evident in large, frequent outbreaks of diphtheria and other vaccine-preventable diseases, straining local health systems.

What is currently needed is a nuanced understanding of the DTP3 performance structure and dynamics over time to elucidate how countries differ in the African region. This can be instrumental in shaping countries’ strategies and future funding policies for global health initiatives, as such data offer utility beyond year-to-year DTP3 coverage levels. With the availability of WUENIC data, which already provide repeated observations of DTP3 coverage for individual countries in the WHO African region, such analysis can be conducted.^8^ For example, estimating DTP3 coverage trajectories over time can reveal variations in immunisation system dynamics. Quantifying these trajectories for individual countries can help identify countries with similar DTP3 profiles. Similar methods have been applied in studies on adolescent cigarette smoking^13^ and multimorbidity in primary care,^14^ where longitudinal data are available. Several existing well-established statistical techniques can support this type of performance analysis.^15^ Given the potential role of this evidence in developing targeted policies to improve immunisation efforts, this study aimed to fill this knowledge gap by analysing temporal trajectories and identifying latent patterns in DTP3 coverage across countries in the WHO African region from 2019 to 2024.

## Methods

### Study design

This study was a retrospective analysis of longitudinal DTP3 coverage data for all 47 countries in the WHO African region from 2019 to 2024.

### Data source

DTP3 coverage data were sourced from the 2024 WUENIC, published on 15 July 2025.^8^ WUENIC was selected over raw national administrative estimates for three reasons. First, WUENIC harmonises administrative data with household survey findings and local expert input to produce the most reliable available country-level estimate, thereby reducing the risk of systematic over-reporting, a known limitation of administrative data.^16^ Second, WUENIC is the official data source used for global immunisation monitoring under the IA2030 framework, ensuring comparability and policy relevance.^6^ Third, WUENIC provides a complete, consistent time series for all 47 countries, which is essential for the longitudinal analytical framework applied in this study.^16 17^ WUENIC data are not based on statistical or mathematical modelling.^16^ To produce country-specific estimates, each country’s data are reviewed independently. If the survey data for a country closely match the reported data (within a 10-percentage-point margin), then the reported data are used as the estimate.^16^ If the reported and survey data conflict, but the survey data seem more reliable, the survey data are used as the estimate, with reference to the survey data from other years.^16^ A detailed description of how the estimates are generated is published elsewhere.^16 17^ The WUENIC data comply with the Guidelines for Accurate and Transparent Health Estimates Reporting (GATHER) criteria.^18^

### Variables

Three variables were extracted: country, year and DTP3 coverage.

#### Outcome variable

DTP3 coverage: the percentage of infants who received three doses of DTP3.

#### Independent variable

Year: 2019–2024. The baseline year for this study was 2019, which was the last full year before the COVID-19 pandemic. Using it as a baseline enabled the analysis to account for COVID-19 disruptions across countries.

Country: all 47 countries in the WHO African region.

### Data analysis

This study used a multilayered analytical framework that combined linear annual growth modelling, mixed-effects longitudinal modelling and principal component (PC) analysis (PCA) with k-means clustering to examine DTP3 coverage dynamics from 2019 to 2024 across all 47 countries in the WHO African region. Linear annual growth modelling and mixed-effects modelling were employed for exploratory purposes to evaluate within-country and between-country variations in coverage trajectories.^15 19^ PCA with k-means clustering was used to identify latent patterns across countries.^20^ Although each of these analytical methods is well established, their combined sequence in this study (figure 1) is conceptually innovative. A detailed description of the analytical procedures for each objective is provided in figure 1.

**Figure 1:**
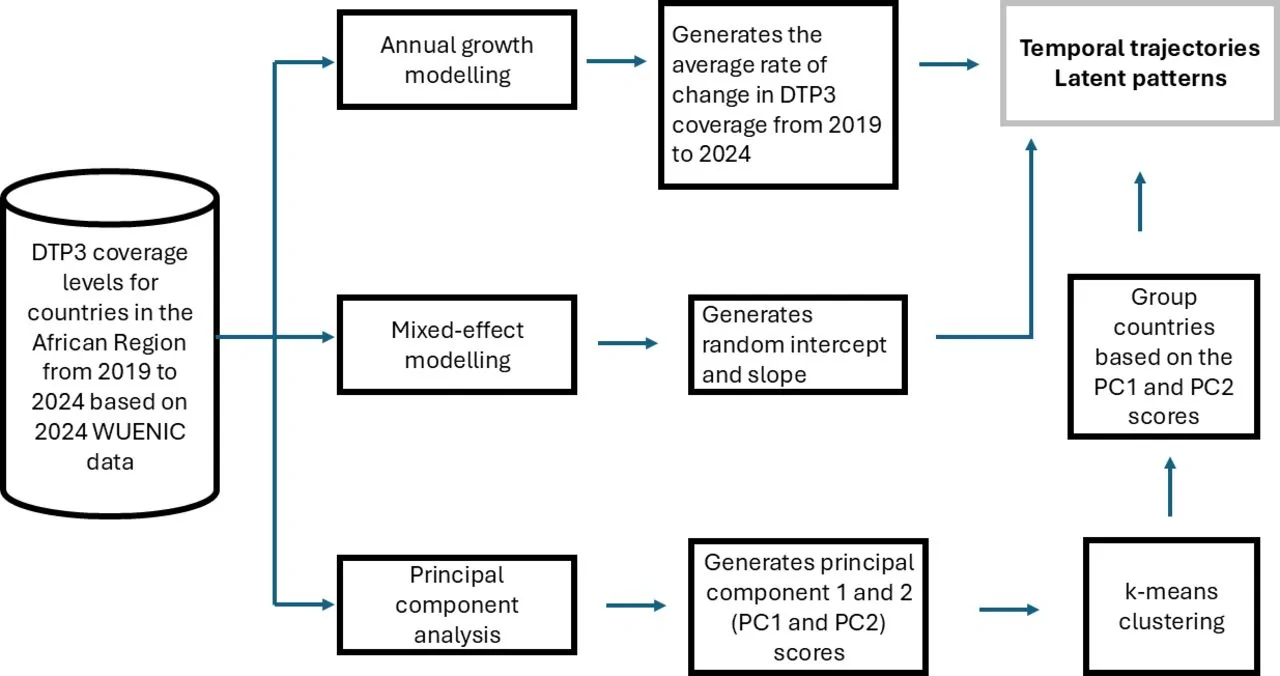
Flow chart of the multilayered analytical framework used in this study. DTP3, diphtheria–tetanus–pertussis-containing vaccines; PC1, principal component 1; PC2, principal component 2; WUENIC, WHO/UNICEF Estimate of National Immunisation Coverage.

#### To explore the temporal trajectories of diphtheria–tetanus–pertussis-containing vaccine coverage across countries in the WHO African region

To measure the annual rate of change in DTP3 coverage from 2019 to 2024 for each of the 47 countries in the WHO African region, a linear annual growth model was fitted using ordinary least squares (OLS) regression, with DTP3 coverage modelled as a function of year for each country separately. These country-specific models estimated slopes representing the average annual change in DTP3 coverage; positive slopes indicated improving coverage and negative slopes indicated decline. A bar chart was created to visualise the direction and magnitude of these slopes. Given that only six annual observations are available per country, slope estimates carry wide 95% CIs and were interpreted as indicative trend directions rather than precise magnitude estimates. The overall rate of change across all countries and the heterogeneity in coverage growth rates between countries were then estimated using a linear mixed-effects model. DTP3 coverage was the dependent variable, and year was the independent variable. 2019 was designated as the baseline year (year 0) to facilitate interpretation of the intercept. The model included both fixed and random effects. The fixed-effect intercept estimated the average DTP3 coverage across all countries at baseline (2019), and the fixed-effect slope estimated the average annual rate of change across the entire region. The random intercept captures between-country differences in baseline DTP3 coverage in 2019, and the random slope captures between-country differences in the annual rate of change. Maximum likelihood estimation was used to estimate all parameters. Model fit was evaluated using three complementary approaches: the likelihood ratio test (LRT) comparing the mixed-effects model to a fixed-effects-only null model, Akaike information criterion (AIC) and the Bayesian information criterion (BIC). The Wald test assessed the statistical significance of fixed effects.

#### To identify latent patterns in diphtheria–tetanus–pertussis-containing vaccine coverage across countries in the WHO African region

Bartlett’s test of sphericity was conducted to determine whether there was sufficient linear correlation in DTP3 coverage per year from 2019 to 2024, and the Kaiser–Meyer–Olkin (KMO) measure of sampling adequacy was calculated to assess suitability for PCA. After these two tests, PCA was performed on the covariance matrix of the 6-year DTP3 coverage data. Component retention was guided by the Kaiser criterion (eigenvalue>1), scree plot and criterion of substantive interpretability. In addition, a PC can be retained based on programmatic relevance. The retained PC scores were subsequently used in a k-means clustering procedure, which grouped each country in the WHO African region into distinct clusters by minimising the sum of squared distances to their respective cluster centroids in the two-dimensional PC space. The number of clusters was set to four after exploring the two-dimensional PC score space. The PC1–PC2 bi-plot reveals four groupings defined by the intersection of sustained coverage level (PC1) and pandemic disruption-and-recovery pattern (PC2), each corresponding to a programmatically distinct archetype. This four-cluster structure was selected for its substantive interpretability within the PC space, which is consistent with the exploratory purpose of this analysis. The k-means algorithm was run without a pre-specified random seed. To verify reproducibility, the analysis was repeated across multiple independent sessions; identical cluster assignments were obtained in all runs, confirming that the four-cluster solution is stable with respect to random initialisation. The countries within each cluster were then reported. Statistical analyses were performed using Stata 14.2.

#### Limitations and strengths of the multilayered analytical framework for diphtheria–tetanus–pertussis-containing vaccine coverage dynamics

The use of linear annual growth modelling enables the calculation of country-specific linear growth rates, transforming static year-to-year coverage data into temporal trajectories (ie, DTP3 coverage change over time). Although this method offers a straightforward measure of coverage change over time, each country is modelled individually; thus, it does not account for cross-country differences. By incorporating mixed-effect modelling, random-effect estimates are generated that capture between-country variation. The PCA with k-means clustering revealed latent structural patterns using an algorithm based on two uncorrelated characteristics.

## Results

### Data availability and use

The latest WUENIC release, published in July 2025, includes data for all 47 countries within the WHO African region. DTP3 coverage levels for all countries from 2019 to 2024 were obtained and used in this study.

### Temporal trajectories of diphtheria–tetanus–pertussis-containing vaccine coverage across countries in the WHO African region

Between 2019 and 2024, DTP3 coverage increased in 21 countries within the WHO African region. The increase was at least 2.0 percentage points in countries such as Chad, Cameroon, Ethiopia, Guinea, Liberia and South Sudan. Liberia experienced a 3.51 percentage-point increase. The percentage-point increases in Chad, Ethiopia, South Sudan, Cameroon and Guinea (listed in descending order) were 3.29, 2.40, 2.34, 2.03 and 2.0, respectively. Meanwhile, over the same period, DTP3 coverage declined in 24 countries in the region. The five largest point-estimated annual declines were recorded in Namibia (3.00 percentage points per year), Mozambique (2.26), São Tomé and Príncipe (2.06), South Africa (2.03) and the Democratic Republic of the Congo (2.00), listed in descending order of magnitude. Of these, São Tomé and Príncipe and the Democratic Republic of the Congo reached statistical significance (online supplemental table S1), whereas the remaining three countries, Namibia (95% CI −6.53 to +0.53), Mozambique (95% CI −9.89 to +5.38) and South Africa (95% CI −4.10 to +0.04) did not. No significant linear change in DTP3 coverage was identified in Eritrea and Burkina Faso during this period. Figure 2 shows the linear changes in DTP3 coverage across all 47 countries. Details regarding CIs and p values for each slope are provided in online supplemental table S1.

10.1136/bmjph-2025-004748.supp1 Supplementary data

**Figure 2:**
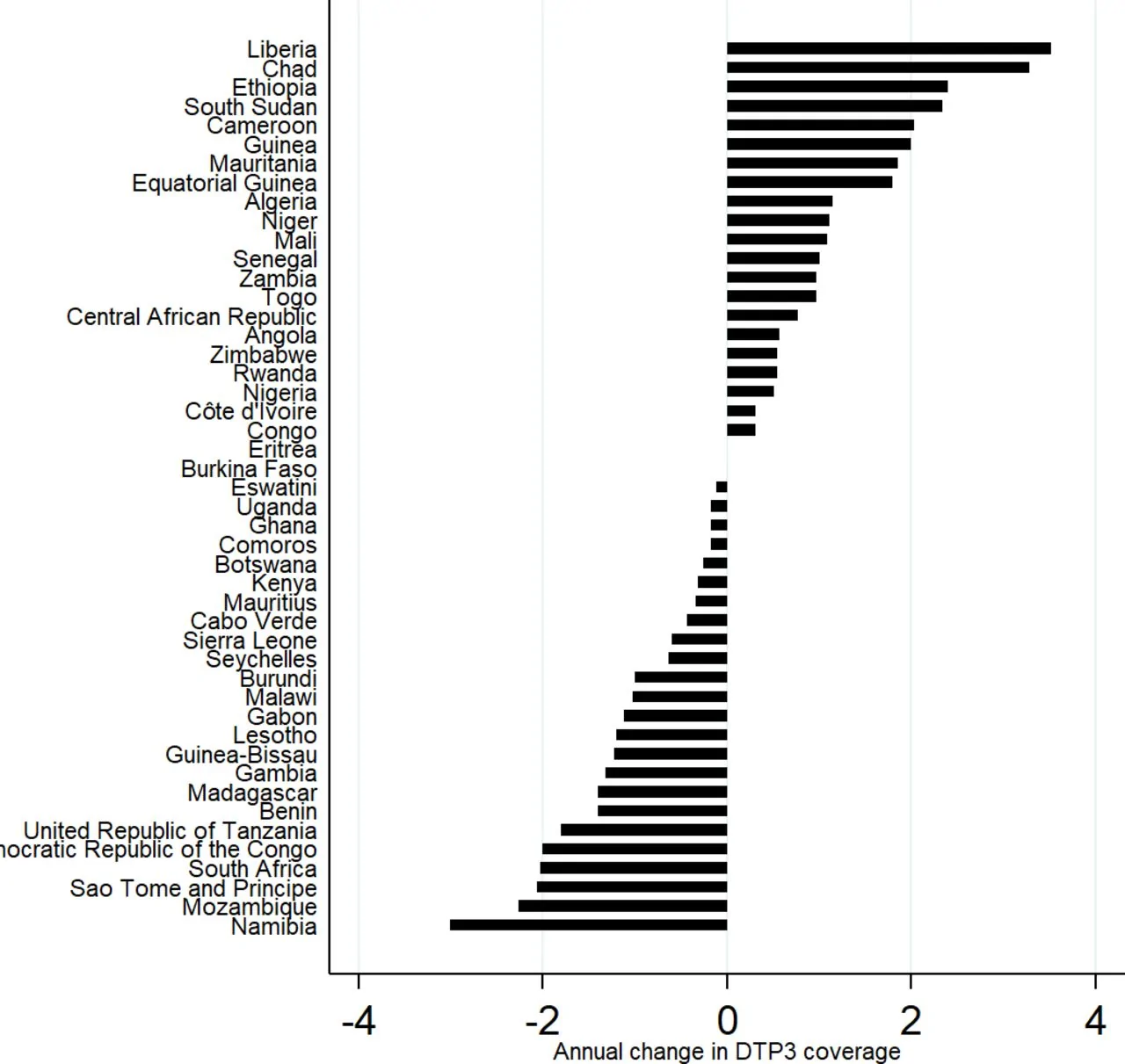
Estimated annual change in DTP3 coverage from 2019 to 2024 for all 47 countries in the WHO African region. DTP3, diphtheria–tetanus–pertussis-containing vaccine.

In the mixed-effects model, the fixed-effect intercept was 79.96 (95% CI 75.97 to 83.96; p<0.001), representing the grand mean of country-specific OLS-fitted values at baseline. The fixed-effect slope was 0.07 percentage points per year (95% CI −0.34 to 0.47; p=0.75), indicating no statistically significant net change in DTP3 coverage across the region over the study period. However, random-effect estimates revealed substantial between-country heterogeneity, with a random-intercept variance of 188.18 and a random-slope variance of 1.18, underscoring marked differences in both baseline coverage levels and rates of change across countries. The substantially larger random-intercept variance relative to the residual variance (13.77) indicates that most unexplained variability in DTP3 coverage resides between countries rather than within countries over time. The AIC was 1801.17, and the BIC was 1819.38. The likelihood ratio test confirmed a significantly better fit of the mixed-effects model over a fixed-effects-only specification (χ²(3) = 494.9, p<0.001).

### Latent patterns in diphtheria–tetanus–pertussis-containing vaccine coverage across countries in the WHO African region

The χ2 from Bartlett’s test of sphericity was 533.19 (p<0.01). KMO measure of sampling adequacy was 0.87. The PCA yielded six components as shown in online supplemental table S2. The eigenvalues for PC1 and PC2 were 5.57 (with a proportion of 0.93) and 0.2 (with a proportion of 0.03), respectively. Based on these two components, scores were computed for each of the 47 countries in the African region. PC1 scores were positive in 27 countries but negative in 20, as illustrated in figure 3. PC2 scores were positive in 23 countries but negative in 24, as shown in figure 4. Based on the PC1 and PC2 scores, four latent patterns in DTP3 coverage were identified (online supplemental table S3). In cluster 1 countries, the average DTP3 coverage per year from 2019 to 2024 ranged from 94.87 to 92.00. In cluster 2, the average DTP3 coverage per year from 2019 to 2024 ranged from 57.22 to 62.78. In cluster 3, the average DTP3 coverage per year from 2019 to 2024 ranged from 69.64 to 75.00. In cluster 4, the average DTP3 coverage per year from 2019 to 2024 ranged from 84.67 to 87.08. Details are provided in online supplemental table S4. The country composition within each cluster, as shown in figure 5, is as follows: cluster 1 includes Botswana, Burkina Faso, Burundi, Cabo Verde, Eritrea, Ghana, Kenya, Lesotho, Malawi, Mauritius, Rwanda, São Tomé and Príncipe, Seychelles, Sierra Leone and Uganda; cluster 2 includes Angola, Central African Republic, Chad, Democratic Republic of the Congo, Ethiopia, Gabon, Guinea, Madagascar and Nigeria; cluster 3 includes Benin, Cameroon, Comoros, Congo, Côte d’Ivoire, Equatorial Guinea, Guinea-Bissau, Liberia, Mali, Mozambique and South Sudan; and cluster 4 includes Algeria, Eswatini, The Gambia, Mauritania, Namibia, Niger, Senegal, South Africa, Togo, the United Republic of Tanzania, Zambia and Zimbabwe.

**Figure 3:**
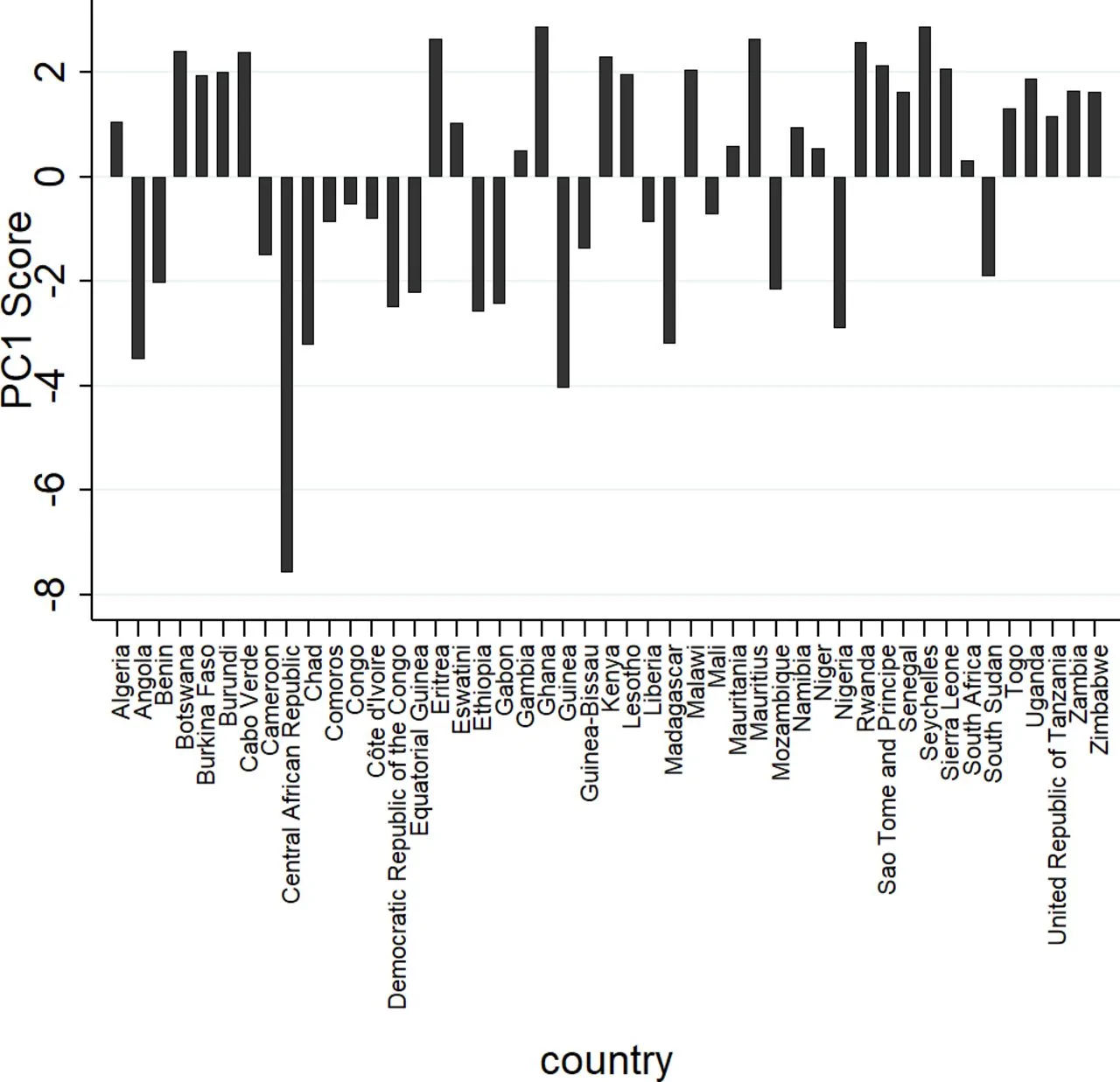
Principal component 1 scores for countries in the WHO African Region. PC1, principal component 1.

**Figure 4:**
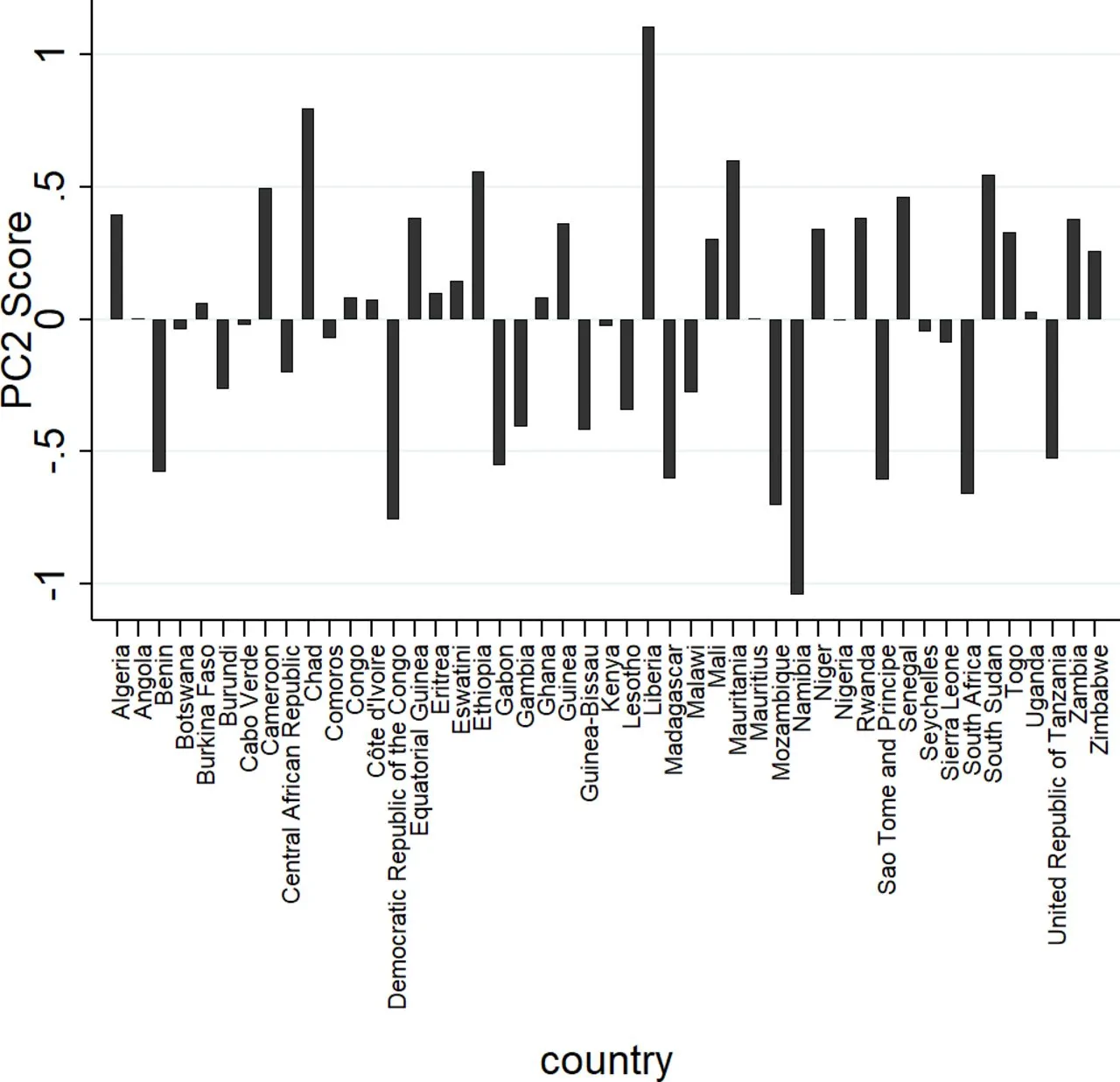
Principal component 2 scores for countries in the WHO African Region. PC2, principal component 2.

**Figure 5:**
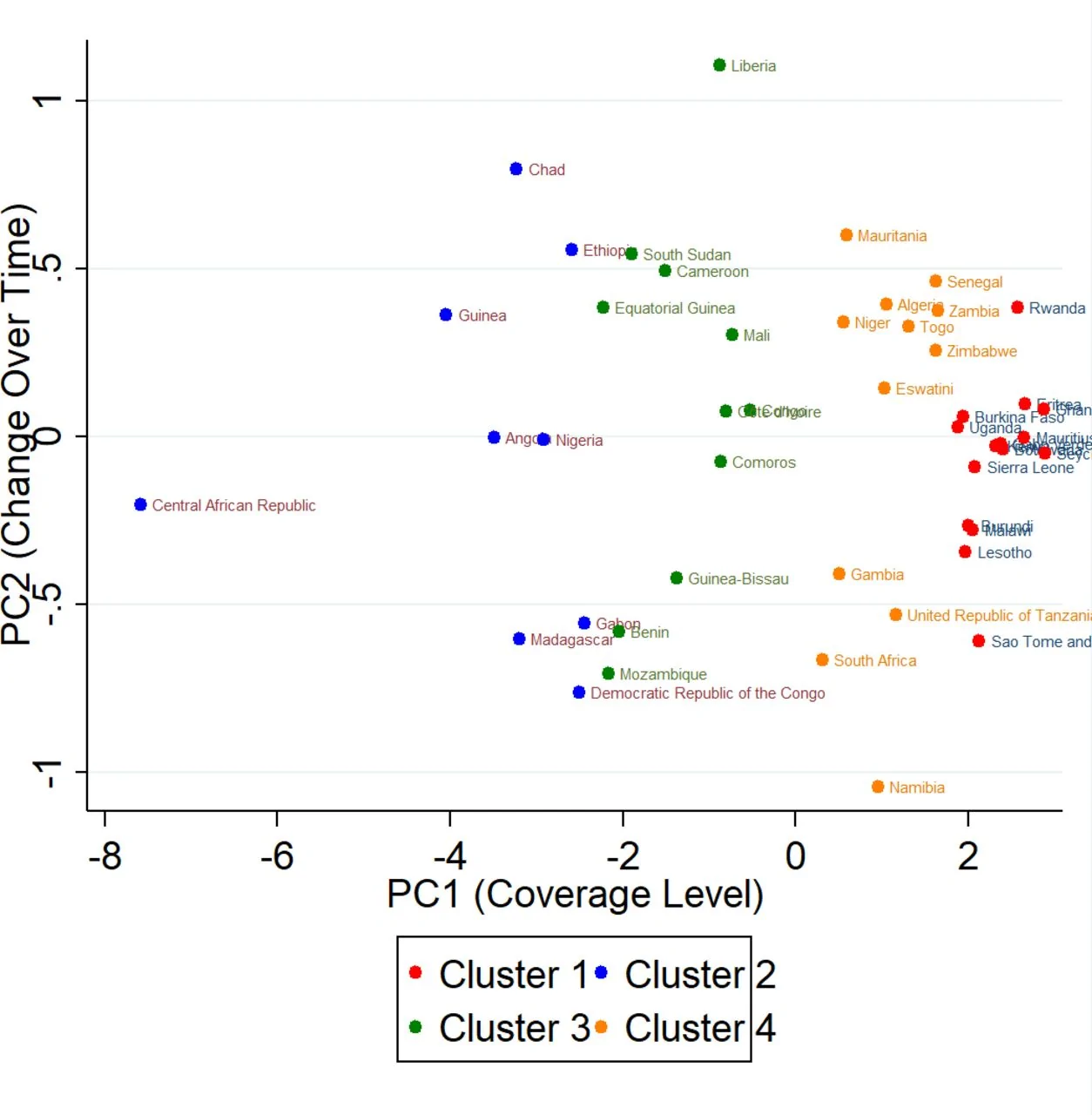
Latent patterns of DTP3 coverage based on principal component scores for countries in the WHO African Region. DTP3, diphtheria–tetanus–pertussis-containing vaccine; PC1, principal component 1; PC2, principal component 2.

## Discussion

This study aimed to explore temporal trajectories and identify patterns in DTP3 coverage across countries in the WHO African region from 2019 to 2024. It found that between 2019 and 2024, DTP3 coverage increased annually by more than 2.0 percentage points in Chad, Cameroon, Ethiopia, Guinea, Liberia and South Sudan. Liberia experienced the highest growth rate at 3.51 percentage points per year. Conversely, DTP3 coverage showed point-estimated annual declines exceeding 2.0 percentage points in the Democratic Republic of the Congo, Mozambique, Namibia, São Tomé and Príncipe and South Africa, with Namibia recording the largest point estimate at −3.0 percentage points per year. The declines in the Democratic Republic of the Congo and São Tomé and Príncipe were statistically significant (p<0.05), whereas those in Namibia, Mozambique and South Africa were not. Eritrea and Burkina Faso maintained DTP3 coverage at 95% and 91%, respectively, with no change over the entire period studied. Furthermore, PCA revealed that two components accounted for most of the variation in DTP3 coverage across the 47 countries from 2019 to 2024. These components enabled the identification of four distinct patterns in DTP3 coverage performance across countries in the WHO African region.

This study quantified the temporal trajectory of DTP3 coverage from 2019 to 2024 for each country in the WHO African region using a single coefficient, offering a parsimonious yet comprehensive measure of programme performance. This analytical paradigm has also been employed in a study in Brazil to examine trends in vaccination coverage over time.^21^ In real-world programme contexts, slopes (the average rate of change over a specified period) derived from linear growth modelling can be practically useful when combined with traditional year-to-year DTP3 coverage levels. While DTP3 coverage levels indicate whether countries have met or maintained the target, they can obscure subtle relative variations. Conversely, slopes reflect the underlying speed and direction of coverage change, serving as proxies for momentum, efficiency and adaptive capacity. Therefore, although DTP3 coverage trajectories are exploratory, they can serve as a higher-order measure of immunisation programme performance.

Several studies examining vaccination coverage before and during the COVID-19 pandemic have reported declining levels of DTP3 and other antigens in some African countries.^22 23^ This study contributes to the existing literature by analysing the trajectory of DTP3 coverage over a longer period, from 2019 (the year before the COVID-19 pandemic) to 2024 (a year after the pandemic ended), providing concrete insights into immunisation programme performance dynamics. As anticipated, the variation in DTP3 coverage across African countries revealed varying trajectories. In 2019, Chad and Liberia had DTP3 coverage of 53% and 71%, respectively. Although suboptimal, these countries experienced the largest increases in the annual rate of change between 2019 and 2024, at 3.29 and 3.51 percentage points, respectively, despite the pandemic, reaching DTP3 coverage of 68% and 83%, respectively, in 2024. Similar trajectories were observed in Ethiopia, Cameroon, Guinea and South Sudan.

Declining trajectories in countries with suboptimal baseline DTP3 coverage are a major concern. Democratic Republic of the Congo, with 73% coverage in 2019, recorded an annual rate of change of −2.00 percentage points. South Africa, with 85% coverage in 2019, recorded an annual rate of change of −2.03 percentage points per year (95% CI −4.10 to 0.04; p=0.053). Mozambique also recorded an annual rate of change of −2.26 percentage points per year (95% CI −9.89 to 5.38; p=0.458). Although these are not statistically significant, they are directionally consistent with the observed raw data. Mozambique’s sharp V-shaped coverage pattern (85% in 2019, declining to 56% in 2021 before partially recovering to 70% in 2024) is worth noting. Overall, these are potentially suggestive of systemic problems distinct from those experienced in other low coverage settings.

Routine monitoring of coverage trajectories can serve as an early warning system even for countries with optimal baseline coverage. For instance, Ghana and Kenya, which have consistently maintained high coverage levels, have shown a modest decline in recent years, warranting a proactive programmatic review. Additionally, countries such as Burundi and Tanzania, which had high DTP3 coverage at baseline (93% and 91%, respectively), recorded negative annual rates of change over the study period (−1.00 and −1.80 percentage points per year, respectively).

A key methodological contribution of this study is the application of PCA to reduce the 6-year DTP3 coverage matrix to two orthogonal dimensions prior to clustering, thereby avoiding the instability that arises from applying k-means directly to high-dimensional, multicollinear coverage data. PC1 (eigenvalue, 5.57; proportion of variance, 0.93) predominantly captured between-country differences in sustained coverage level across the study period. Countries with positive PC1 scores, such as Botswana, Burkina Faso and Burundi, performed above the multi-year regional mean, whereas countries with negative scores, including Nigeria, the Democratic Republic of the Congo and Ethiopia, performed below it. Critically, countries with consistently low but improving coverage, such as Chad and Liberia, are assigned negative PC1 scores regardless of their upward momentum, underscoring the necessity of PC2.

PC2 (eigenvalue, 0.20; proportion of variance, 0.03) captures the contrast between early-pandemic coverage and late-period coverage, reflecting pandemic disruption and post-pandemic recovery trajectories relative to the pre-pandemic baseline. Countries with high PC2 scores had stronger late-period coverage relative to their early-period baseline, and those with low PC2 scores maintained higher early-period coverage but saw late-period erosion. Chad and Liberia held the highest PC2 scores. Chad experienced sustained improvement across all 6 years, and Liberia’s strong post-2021 rebound both produced high late-period relative to early-period coverage. Namibia and the Democratic Republic of the Congo held the lowest PC2 scores, reflecting persistent late-period decline.

By combining the coverage-level dimension (PC1) and the trajectory dimension (PC2) within a k-means algorithm, this study derived a two-dimensional performance space in which countries are positioned relative to one another and assigned to the nearest cluster centroid. The resulting four archetypes each carry distinct programmatic implications. Even within the four archetypes, there is observed within-cluster heterogeneity; as such, it is important to note that cluster labels describe the dominant aggregate profile of each archetype. Two countries in particular, São Tomé and Príncipe (cluster 1) and Mozambique (cluster 3), are within-cluster outliers.

Cluster 1 (n=15; 31.91%) is the largest cluster and represents countries with the highest sustained DTP3 coverage in the region (average 92%–95% across the study period). This cluster is characterised by high positive PC1 scores and near-zero PC2 scores. Cluster 2 (n=9; 19.15%) is defined by the lowest sustained PC1 scores in the region, corresponding to average DTP3 coverage below 70% across the study period. The cluster’s defining characteristic is chronically low absolute coverage. Cluster 3 (n=11; 23.40%) is defined by below-average PC1 scores and above-average PC2 scores. At the cluster level, the average DTP3 coverage across cluster 3 countries rose from 73.55% in 2019 to 74.00% in 2024, indicating a broadly positive aggregate trend. Cluster 4 (n=12; 25.53%) warrants careful interpretation. Although cluster 4 countries have higher average absolute DTP3 coverage (84%–87%) than cluster 3 countries (69%–75%), they are characterised by moderate-to-high PC1 scores coupled with neutral-to-negative PC2 scores.

### Limitations of the study

The findings of this study should be interpreted in the context of several limitations. First, WUENIC estimates are derived from triangulation of administrative data, household surveys, and local expert opinion. Although WUENIC applies rigorous quality criteria and is the most reliable available source for this purpose, the estimates remain subject to limitations in the accuracy and completeness of the underlying data. Administrative data are prone to over-reporting, while survey data are subject to recall bias and infrequent collection cycles in many African countries. Second, the analysis was restricted to DTP3 coverage. While DTP3 is the primary infant immunisation indicator under IA2030 Impact Goal 3.1, it does not capture the full spectrum of immunisation programme performance. It does not measure the DTP1-to-DTP3 dropout rate, which reflects the inability to retain children already in the immunisation system. Nor does it capture second-year-of-life vaccines, such as the second dose of the measles-containing vaccine (MCV2), which would likely alter the cluster configuration in some countries. Including additional antigens (eg, measles, PCV and rotavirus) or composite indicators in future analyses would provide a more comprehensive characterisation of immunisation system performance. Third, the linear growth model assumes a constant rate of change over the study period, which does not adequately capture non-linear dynamics in countries whose trajectories include a distinct pandemic disruption phase (2019–2021) followed by a recovery phase (2021–2024) with a different direction or steepness. In future, when more data points are available, a piecewise or segmented regression approach should be considered. Fourth, no explanatory covariates were included in this analysis. This was a deliberate design decision as the primary goal was to characterise the structure and dynamics of DTP3 coverage. Including macro-level predictors such as gross domestic product, health expenditure, political stability or conflict indices at this stage without a clear theoretical model risks ecological fallacy and reductionist interpretation of what are known to be highly contextual and multifactorial determinants.^24–28^ Future studies should incorporate such predictors within multilevel frameworks guided by the cluster typology derived here to develop a mechanistic understanding of the drivers of each archetype.

### Implications for policy and practice

Quantifying DTP3 performance across countries in the African region and classifying them based on shared characteristics, as demonstrated in this study, can help guide decision-making, including funding policies. In cluster 1 (high coverage, consolidating), the primary risk is slow attrition. The priority is therefore close monitoring and early warning, strengthening digital immunisation registries and information systems to detect and respond to sub-national coverage gaps before they aggregate into national erosion. Workforce retention, supply chain reliability and knowledge transfer to peers in cluster 3 are additional priorities. In cluster 2 (chronically low coverage, divergent trajectories), a single uniform strategy is insufficient because the cluster contains fundamentally different sub-types. For rapidly improving members such as Chad and Ethiopia, the priority is context-specific acceleration, identifying and protecting the specific programme elements driving gains, and investing to close the remaining gap. For cluster 3 countries, the strategic priority is consolidation, scale-up and sustainability assurance. This involves identifying precisely which interventions, delivery platforms or governance reforms have driven the observed improvements, investing in immunisation information systems to sustain data quality and supporting domestic financing transitions to reduce aid dependency. For cluster 4 countries, a dual approach is warranted: an immediate diagnostic phase to identify the specific drivers of declining trajectories through rapid programme reviews, followed by targeted stabilisation investments in the identified bottlenecks, such as workforce gaps, supply chain failures, declining community demand or fiscal constraints. A detailed description of recommended actions is provided in online supplemental table S5.

### Implications for future implementation research

The cluster typology generated by this study provides a structured framework to guide targeted implementation research for each archetype. A primary priority is developing a context-specific, mechanistic understanding of why countries within the same cluster share performance characteristics and why countries in different clusters diverge. Such inquiry should be anchored in core implementation science frameworks such as the Consolidated Framework for Implementation Research^29^ and broader Health Systems Framework^30^ and should encompass health system financing, governance and accountability mechanisms, political economy, health workforce capacity and distribution, vaccine supply chain architecture, demand generation strategies and integration with primary healthcare platforms. Mixed methods design that pairs routine data analysis with qualitative inquiry, including document reviews of national EPI programme reports, key informant interviews and systems mapping, is well-suited to this purpose and will yield contextual depth that quantitative analysis alone cannot provide.

## Conclusion

This study quantified, at the individual-country level, the speed, direction and structural patterning of DTP3 coverage trajectories across the WHO African region from 2019 to 2024. By applying a multilayered analytical framework integrating linear growth modelling, mixed-effects modelling and PCA-informed k-means clustering, it generated an empirically grounded typology of four immunisation system archetypes that extends beyond point-in-time coverage levels to capture both performance status and momentum. These archetypes provide a principled basis for segmenting immunisation policy and investment across the region, enabling decision-makers to move from uniform, region-wide prescriptive strategies to differentiated, archetype-specific programmatic strategies. The typology also establishes a phenotypic foundation for a future agenda of implementation research aimed at explaining differential progress and rigorously testing context-sensitive innovations to accelerate DTP3 coverage towards the IA2030 target of 90% across Africa.

## Data Availability

Data are available in a public, open access repository.

## References

[1] Shattock AJ, Johnson HC, Sim SY, et al. Contribution of vaccination to improved survival and health: modelling 50 years of the Expanded Programme on Immunization. The Lancet 2024;403:2307–16. 10.1016/S0140-6736(24)00850-X PMC11140691 38705159

[2] Boutayeb A. The impact of infectious diseases on the development of africa. In: Handbook of disease burdens and quality of life measures. Springer, 2010: 1171–88.

[3] Michaud CM. Global burden of infectious diseases. Encyclopedia of Microbiology 2009. 10.1016/B978-012373944-5.00185-1

[4] Basu RN. Expanded programme on immunization and primary health care. J Commun Dis 1982;14:183–8. 7183716

[5] Henderson RH. The Expanded Programme on Immunization of the World Health Organization. Rev Infect Dis 1984;6 Suppl 2:S475–9. 10.1093/clinids/6.supplement_2.s475 6740096

[6] Immunization Agenda 2030 Partners. Immunization agenda 2030: A global strategy to leave no one behind. Vaccine 2024;203042:s5–14. 10.1016/j.vaccine.2022.11.042 39004466

[7] World Heath Organization. WHO. World Health Organization; 2023. Available: https://apps.who.int/gho/data/view.main-afro.81200?lang=en

[8] World Health Organization. 2024 WHO/UNICEF estimates of national immunization coverage (WUENIC). 2024. Available: https://www.who.int/publications/m/item/progress-and-challenges

[9] Kim S, Headley TY, Tozan Y. Universal healthcare coverage and health service delivery before and during the COVID-19 pandemic: A difference-in-difference study of childhood immunization coverage from 195 countries. PLoS Med 2022;19:e1004060. 10.1371/journal.pmed.1004060 PMC9380914 35972985

[10] Aguinaga-Ontoso I, Guillen-Aguinaga S, Guillen-Aguinaga L, et al. n.d. COVID-19 Impact on DTP Vaccination Trends in Africa: A Joinpoint Regression Analysis. Vaccines (Basel) 11:1103. 10.3390/vaccines11061103 PMC10305165 37376492

[11] O’Brien KL, Lemango E. The big catch-up in immunisation coverage after the COVID-19 pandemic: progress and challenges to achieving equitable recovery. The Lancet 2023;402:510–2. 10.1016/S0140-6736(23)01468-X 37478887

[12] Collins Mwai, VaccinesWork. One year of the big catch-up: what progress has been made? 2024. Available: https://www.gavi.org/vaccineswork/one-year-big-catch-what-progress

[13] Audrain-McGovern J, Rodriguez D, Tercyak KP, et al. Identifying and Characterizing Adolescent Smoking Trajectories. Cancer Epidemiology, Biomarkers & Prevention 2004;13:2023–34. 10.1158/1055-9965.2023.13.12 15598757

[14] Strauss VY, Jones PW, Kadam UT, et al. Distinct trajectories of multimorbidity in primary care were identified using latent class growth analysis. J Clin Epidemiol 2014;67:1163–71. 10.1016/j.jclinepi.2014.06.003 PMC4165436 25063556

[15] Herle M, Micali N, Abdulkadir M, et al. Identifying typical trajectories in longitudinal data: modelling strategies and interpretations. Eur J Epidemiol 2020;35:205–22. 10.1007/s10654-020-00615-6 PMC7154024 32140937

[16] Burton A, Monasch R, Lautenbach B, et al. WHO and UNICEF estimates of national infant immunization coverage: methods and processes. Bull World Health Organ 2009;87:535–41. 10.2471/blt.08.053819 PMC2704038 19649368

[17] Burton A, Kowalski R, Gacic-Dobo M, et al. A formal representation of the WHO and UNICEF estimates of national immunization coverage: a computational logic approach. PLoS One 2012;7:e47806. 10.1371/journal.pone.0047806 PMC3485034 23133527

[18] Danovaro-Holliday MC, Gacic-Dobo M, Diallo MS, et al. Compliance of WHO and UNICEF estimates of national immunization coverage (WUENIC) with Guidelines for Accurate and Transparent Health Estimates Reporting (GATHER) criteria. Gates Open Res 2021;5:77. 10.12688/gatesopenres.13258.1 PMC9021403 35615619

[19] Curran PJ, Bauer DJ. The disaggregation of within-person and between-person effects in longitudinal models of change. Annu Rev Psychol 2011;62:583–619. 10.1146/annurev.psych.093008.100356 PMC3059070 19575624

[20] Ding C, He X. k -means clustering via principal component analysis. Twenty-first international conference; Banff, Alberta, Canada, 2004:29. 10.1145/1015330.1015408 Available: http://portal.acm.org/citation.cfm?doid=1015330

[21] Neves ABB, Silva LE de O, Amaral GMC, et al. Temporal trends in vaccination coverage in the first year of life in Brazil. Rev paul pediatr 2023;42:e2023020. 10.1590/1984-0462/2024/42/2023020

[22] Masresha BG, Luce Jr R, Shibeshi ME, et al. The performance of routine immunization in selected African countries during the first six months of the COVID-19 pandemic. Pan Afr Med J 2020;37. 10.11604/pamj.supp.2020.37.1.26107 PMC7733346 33343791

[23] Mboussou F, Kada S, Danovaro-Holliday MC, et al. Status of Routine Immunization Coverage in the World Health Organization African Region Three Years into the COVID-19 Pandemic. Vaccines (Basel) 2024;12:2. 10.3390/vaccines12020168 PMC10891650 38400151

[24] Matthew Ayodele A, Fasasi MI, Rejoice Uche O, et al. Factors associated with full childhood vaccination coverage among young mothers in Northern Nigeria. Pan Afr Med J 2024;47:4. 10.11604/pamj.2024.47.4.37517 PMC10870161 38371647

[25] Mwamba GN, Yoloyolo N, Masembe Y, et al. Vaccination coverage and factors influencing routine vaccination status in 12 high risk health zones in the Province of Kinshasa City, Democratic Republic of Congo (DRC), 2015. Pan Afr Med J 2017;27:7. 10.11604/pamj.supp.2017.27.3.11930 PMC5745950 29296142

[26] Galadima AN, Zulkefli NAM, Said SM, et al. Factors influencing childhood immunisation uptake in Africa: a systematic review. BMC Public Health 2021;21::1475. 10.1186/s12889-021-11466-5 PMC8320032 34320942

[27] Eze P, Agu UJ, Aniebo CL, et al. Factors associated with incomplete immunisation in children aged 12-23 months at subnational level, Nigeria: a cross-sectional study. BMJ Open 2021;11:e047445. 10.1136/bmjopen-2020-047445 PMC8237740 34172548

[28] Mihigo R, Okeibunor J, Anya B, et al. Challenges of immunization in the African Region. Pan Afr Med J 2017;27:12. 10.11604/pamj.supp.2017.27.3.12127 PMC5745929 29296147

[29] Damschroder LJ, Reardon CM, Widerquist MAO, et al. The updated Consolidated Framework for Implementation Research based on user feedback. Implement Sci 2022;17:75. 10.1186/s13012-022-01245-0 PMC9617234 36309746

[30] Organization WH. Everybody’s business: strengthening health systems to improve health outcomes: who’s framework for action. 2007.

