# Supplementary material for: Infant vaccination coverage trajectories and immunisation system archetypes in the WHO African region, 2019–2024: a longitudinal analysis of 47 countries
Source: BMJ Public Health. 2026 Oct 12;4(Suppl 1):e004748. doi: 10.1136/bmjph-2025-004748 (PMC13662025; doi:10.1136/bmjph-2025-004748)
Supplement: Supplementary file 1 [file bmjph-4-Suppl_1-s001.pdf]

Supplementary file

Table S1: Trajectories of DTP3 coverage from 2019 to 2024 for countries in the WHO African Region

Country	Average annual change in DTP3 coverage	95% Confidence Interval	P-Value
Algeria	1.14	-1.84 to 4.13	0.49
Liberia	3.51	1.31 to 5.72	0.04
Sierra Leone	-0.60	-1.20 to -0.00	0.12
Angola	0.57	-2.03 to 3.18	0.69
Benin	-1.40	-2.23 to -0.57	0.03
Botswana	-0.26	-2.17 to 1.65	0.80
Burkina Faso	0.00	-	-
Burundi	-1.00	-1.57 to -0.43	0.03
Cabo Verde	-0.43	-0.91 to 0.06	0.16
Cameroon	2.03	1.59 to 2.47	0.00
Central African Republic	0.77	-0.77 to 2.31	0.38
Chad	3.29	2.29 to 4.28	0.00
Comoros	-0.17	-1.38 to 1.04	0.80
Congo	0.31	-0.76 to 1.39	0.60
Côte d'Ivoire	0.31	-1.34 to 1.97	0.73
Democratic Republic of the Congo	-2.00	-3.35 to -0.65	0.04
Equatorial Guinea	1.80	0.42 to 3.18	0.06
Eritrea	0.00	-	-
Eswatini	-0.11	-3.68 to 3.45	0.95
Ethiopia	2.40	1.34 to 3.46	0.01
Gabon	-1.11	-4.08 to 1.85	0.50
Gambia	-1.31	-2.50 to -0.13	0.10
Ghana	-0.17	-1.19 to 0.84	0.76
Guinea	2.00	1.73 to 2.27	0.00
Guinea-Bissau	-1.23	-3.06 to 0.60	0.26
Kenya	-0.31	-0.94 to 0.31	0.38
Lesotho	-1.20	-2.30 to -0.10	0.10
Madagascar	-1.40	-4.04 to 1.24	0.36
Malawi	-1.03	-2.37 to 0.31	0.21
Mali	1.09	-0.66 to 2.83	0.29
Mauritania	1.86	0.19 to 3.52	0.09
Mauritius	-0.34	-0.81 to 0.12	0.22
Mozambique	-2.26	-7.65 to 3.13	0.46
Namibia	-3.00	-5.49 to -0.51	0.08
Niger	1.11	0.87 to 1.36	0.00
Nigeria	0.51	-1.22 to 2.25	0.59
Rwanda	0.54	-1.63 to 2.72	0.65
Sao Tome and Principe	-2.06	-3.23 to -0.88	0.03
Senegal	1.00	-0.92 to 2.92	0.37
Seychelles	-0.63	-1.45 to 0.20	0.21

Country	Average annual change in DTP3 coverage	95% Confidence Interval	P-Value
South Africa	-2.03	-3.49 to -0.57	0.05
South Sudan	2.34	1.31 to 3.37	0.01
Togo	0.97	0.36 to 1.58	0.04
Uganda	-0.17	-0.94 to 0.60	0.69
United Republic of Tanzania	-1.80	-2.47 to -1.13	0.01
Zambia	0.97	-0.24 to 2.18	0.19
Zimbabwe	0.54	-0.26 to 1.34	0.25

Table S2: Principal components of DTP3 coverage from 2019 to 2024 in the WHO African Region

Component	Eigenvalue	Difference	Proportion	Cumulative
Comp1	5.57	5.37	0.93	0.93
Comp2	0.20	0.09	0.03	0.96
Comp3	0.12	0.05	0.02	0.98
Comp4	0.06	0.03	0.01	0.99
Comp5	0.03	0.01	0.00	1.00
Comp6	0.02	.	0.00	1.00

Table S3: Principal component scores based on DTP3 coverage from 2019 to 2024 for countries in the WHO African Region

Country	Principal Component 1 (PC1) Score	Principal Component 2 (PC2) Scores	Cluster
Rwanda	2.571311	0.3844243	1
Eritrea	2.650676	0.0974808	1
Ghana	2.871885	0.0813096	1
Burkina Faso	1.935909	0.0594246	1
Uganda	1.878821	0.0279578	1
Mauritius	2.648154	-0.0022152	1
Cabo Verde	2.378211	-0.0222292	1
Kenya	2.311869	-0.028547	1
Botswana	2.40152	-0.0377386	1
Seychelles	2.884958	-0.0496502	1
Sierra Leone	2.076809	-0.0888177	1
Burundi	2.00216	-0.2652024	1
Malawi	2.053563	-0.2764941	1
Lesotho	1.966156	-0.3437648	1
Sao Tome and Principe	2.125262	-0.6076565	1
Chad	-3.232809	0.7971417	2
Ethiopia	-2.595535	0.5583521	2

Country	Principal Component 1 (PC1) Score	Principal Component 2 (PC2) Scores	Cluster
Guinea	-4.046062	0.3635097	2
Angola	-3.493262	-0.0016651	2
Nigeria	-2.914359	-0.0072912	2
Central African Republic	-7.589195	-0.2012643	2
Gabon	-2.44931	-0.5537631	2
Madagascar	-3.197898	-0.6031467	2
Democratic Republic of the Congo	-2.505953	-0.7601309	2
Liberia	-0.8826701	1.108415	3
South Sudan	-1.905918	0.5440074	3
Cameroon	-1.510754	0.4955719	3
Equatorial Guinea	-2.23103	0.3847555	3
Mali	-0.7314236	0.3054233	3
Congo	-0.532286	0.0804388	3
Côte d'Ivoire	-0.8091732	0.0750443	3
Comoros	-0.8673837	-0.0735586	3
Guinea-Bissau	-1.375733	-0.4206333	3
Benin	-2.046928	-0.5794923	3
Mozambique	-2.165581	-0.7060018	3
Mauritania	0.5845401	0.6000063	4
Senegal	1.625525	0.4641739	4
Algeria	1.048355	0.3946252	4
Zambia	1.64088	0.3772029	4
Niger	0.5553868	0.3419763	4
Togo	1.304399	0.3303127	4
Zimbabwe	1.61743	0.2563287	4
Eswatini	1.02553	0.1455181	4
Gambia	0.5047678	-0.40944	4
United Republic of Tanzania	1.159915	-0.5298876	4
South Africa	0.3059605	-0.6632797	4
Namibia	0.9533114	-1.04153	4

Table S4: Average DTP3 coverage per year for each cluster

Cluster	DTP3 Coverage 2019	DTP3 Coverage 2020	DTP3 Coverage 2021	DTP3 Coverage 2022	DTP3 Coverage 2023	DTP3 Coverage 2024
1	94.87	93.33	93.73	92.60	92.53	92.00
2	60.78	58.78	58.56	57.22	62.78	62.56
3	73.55	69.91	69.64	72.27	75.00	74.00
4	87.08	85.00	84.67	86.58	86.92	85.17

Table S5: Cluster-specific recommended actions for immunization systems in the WHO African Region

Cluster	Sub-group (PC2-based)	PC2 range	PC2 pattern	Suggested Action	Action type
Cluster 1 High coverage, consolidating n = 15	Sub-group A [PC2 ≥ 0 n = 5 (Rwanda +0.39, Eritrea +0.10, Ghana +0.08, Burkina Faso +0.06, Uganda +0.03)]	+0.03 to +0.39	Stronger late-period recovery relative to early-period	Sub-national zero-dose mapping: Commission DHIS2-integrated district-level dashboards to identify declining wards before national coverage falls below 90%; Rwanda's district dashboard is directly replicable across Sub-group A members<br>Maintain performance and share experience: Rwanda, Ghana, and Uganda have positive PC2 scores indicating sustained late-period recovery; structured South-South exchanges channelling their programme experience to Cluster 3 peers are the priority action<br>Domestic financing consolidation: Integrate DTP3 into multi-year domestic budget lines; Eritrea and Burkina Faso's perfectly constant coverage warrants implementation research to understand what sustains this stability	Surveillance Knowledge transfer Financing
	Sub-group B [(PC2 < 0 n = 10 (São Tomé and Príncipe −0.61 Lesotho −0.35 Malawi −0.28 Burundi −0.27 Sierra Leone −0.09 Seychelles −0.05 Botswana −0.04 Kenya −0.03 Cabo Verde −0.02 Mauritius −0.00)]	−0.61 to −0.00	Late-period coverage below the early-period baseline relative to peers	Biennial EPI programme audits: Systematically review cold chain, health worker attrition, stock-outs, and demand; São Tomé and Príncipe (PC2 = −0.61, the strongest negative recovery pattern in C1) and Lesotho (PC2 = −0.35) are priority candidates	Surveillance Retention Early warning

Cluster	Sub-group (PC2-based)	PC2 range	PC2 pattern	Suggested Action	Action type
				Health worker retention schemes: Non-monetary incentives (performance recognition, career progression pathways, community health officer certification) to curtail frontline attrition in otherwise functional high-coverage systems<br>Attrition early-warning system: Countries near PC2 = 0 (Mauritius, Cabo Verde, Kenya, Botswana) are at the transition point; monitor annually and trigger audit protocol if PC2 turns negative in future WUENIC cycles	
Cluster 2 Chronically low coverage, divergent trajectories n = 9	Sub-group A [PC2 ≥ 0 n = 3 (Chad +0.81, Ethiopia +0.56, Guinea +0.37)]	+0.37 to +0.81	Strong positive recovery pattern relative to regional peers	Implementation research first: Commission process evaluations to identify which programme elements drive the strong positive PC2 patterns; Chad's Gavi-supported outreach and Ethiopia's Health Extension Worker programme are candidate mechanisms<br>Acceleration investment: Address specific bottlenecks: cold chain last-mile logistics, outreach frequency in underserved zones, integration with maternal and child health contacts; protect gains from Gavi transition risk through domestic budget lines<br>South-South learning: Chad (PC2 = +0.81) and Ethiopia (PC2 = +0.56) are the strongest late-period recovery stories in the chronically low-coverage group; document and disseminate their programme innovations to Sub-group B peers	Research<br>Acceleration<br>Financing<br>Knowledge
	Sub-group B [PC2 < 0 n = 6 (DRC −0.77, Madagascar −0.61, Gabon −0.56, CAR −0.20, Nigeria −0.01, Angola −0.00)]	−0.77 to −0.00	Late-period coverage did not recover relative to the early period	Conflict-sensitive EPI (DRC, Madagascar): Strongest negative PC2 in C2; negotiate humanitarian access corridors, deploy mobile teams with GPS-tracked cold boxes, embed immunisation into emergency response and disaster risk reduction plans	Emergency<br>Diagnosis<br>Monitoring

Cluster	Sub-group (PC2-based)	PC2 range	PC2 pattern	Suggested Action	Action type
				Bottleneck analysis before investment (Gabon): Gabon is upper-middle income with a negative PC2 despite adequate resources; commission bottleneck analysis to diagnose whether failure reflects access, supply, utilisation, or governance gaps<br>Monitor borderline cases (Nigeria, Angola): Both have PC2 ≈ 0, indicating marginal late-period performance; they are not in acute decline but require regular monitoring.	
Cluster 3 Mid-low coverage, predominantly improving n = 11	Sub-group A [PC2 ≥ 0 n = 7 (Liberia +1.12 S. Sudan +0.55 Cameroon +0.50 Eq. Guinea +0.39 Mali +0.31 Congo +0.08 Côte d’Ivoire +0.08)]	+0.08 to +1.12	Positive recovery pattern; late-period gains	Consolidate CHW outreach into government systems<br>EPI–PHC integration: Establish shared registers, joint outreach, and combined supply chains between EPI and antenatal care, nutrition, and well-child contacts so every existing service contact becomes an immunisation opportunity<br>Demand generation: Deploy community radio and SMS reminder systems linked to birth registration databases; Congo and Côte d’Ivoire (PC2 ≈ +0.08) are borderline and need demand-side support to sustain positive trajectory	Consolidation Integration Demand
	Sub-group B [PC2 < 0 n = 4 (Mozambique −0.71, Benin −0.59, Guinea-Bissau −0.43, Comoros −0.07)]	−0.71 to −0.07	Late-period coverage below the early-period baseline relative to peers	Shock-adaptive EPI redesign (Mozambique): Strongest negative PC2 in C3 (−0.71); coverage collapsed 85%→70% under concurrent climate disasters and the Cabo Delgado insurgency; redesign as shock-adaptive: pre-positioned vaccines, disaster-triggered protocols, immunisation integrated into the national multi-hazard early warning system	Resilience Recovery Monitoring

Cluster	Sub-group (PC2-based)	PC2 range	PC2 pattern	Suggested Action	Action type
				Costed 12-month recovery (Benin, Guinea-Bissau): PC2 = −0.59 and −0.43,respectively; commission bottleneck analyses and develop costed recovery plans with quarterly milestones; Guinea-Bissau's chronic political instability requires a governance-sensitive design<br>Monitor Comoros (PC2 = −0.07): Marginally negative PC2; not in acute crisis but at risk of further erosion; annual monitoring with a pre-specified trigger for full bottleneck review if PC2 worsens	
Cluster 4 Mid-high coverage, erosion risk n = 12	Sub-group A [PC2 ≥ 0 n = 8 (Mauritania +0.61, Senegal +0.47 Algeria +0.40 Zambia +0.38 Niger +0.35 Togo +0.33 Zimbabwe +0.26 Eswatini +0.15)]	+0.15 to +0.61	Positive recovery pattern; late-period stability or improvement	Last-mile equity gap closure: Algeria (PC2 = +0.40) and Senegal (PC2 = +0.47) are approaching or at the IA2030 90% threshold; address nomadic and remote populations through motorcycle or camel cold chain and seasonally aligned outreach campaigns<br>Civil registration–immunisation linkage: Strengthen record linkage to improve denominator accuracy and enable child-level default tracking; Zimbabwe and Zambia have existing civil registration infrastructure available for integration<br>Monitor Eswatini (PC2 = +0.15): Positive PC2 but erratic annual pattern (90%→83%→77%→97%→85%→84%); the positive PC2 reflects 2022–2024 values relative to 2019–2020,but the year-to-year instability warrants a cold chain and data quality audit	Equity Data systems Monitoring
	Sub-group B [PC2 < 0 n = 4 (Namibia −1.05, South Africa −0.67, Tanzania	−1.05 to −0.41	Strongest late-period erosion pattern in the region	Emergency supply chain diagnostic (Namibia): Strongest negative PC2 in C4 and in the entire dataset (−1.05); coverage rose 87%→94% then collapsed to 74%; commission district-level	Emergency Integration Triage Exchange

Cluster	Sub-group (PC2-based)	PC2 range	PC2 pattern	Suggested Action	Action type
	−0.54, Gambia −0.41)]			diagnostic within 6 months and implement findings before further erosion compounds	
				Mandatory private-sector data integration (South Africa): PC2 = −0.67; decline from 85% to 74% in a dual public-private system; mandate routine reporting from the entire system, and design targeted outreach for informal settlement populations	
				Sub-national triage (Tanzania): PC2 = −0.54; steady decline 91%→83% across a large decentralised country; rank all districts by 2019–2024 pattern to identify whether decline is geographically concentrated before designing district-specific recovery plans	
				Bilateral programme exchanges (Gambia): PC2 = −0.41; pair with a Cluster 1 high-performer for structured secondments and implementation documentation transfer; a high-value, low-cost mechanism appropriate for a small-country context	
